# Postprandial lipemic and inflammatory responses to high-fat meals: a review of the roles of acute and chronic exercise

**DOI:** 10.1186/s12986-016-0142-6

**Published:** 2016-11-16

**Authors:** Colby S. Teeman, Stephanie P. Kurti, Brooke J. Cull, Sam R. Emerson, Mark D. Haub, Sara K. Rosenkranz

**Affiliations:** 1 Department of Food, Nutrition, Dietetics and Health, Kansas State University, 212 Justin Hall, 1324 Lovers Lane, 66506 Manhattan, KS USA; 2 Department of Kinesiology, Kansas State University, 1A Natatorium, 920 Denison Ave, 66506 Manhattan, KS USA; 3 Physical Activity and Nutrition-Clinical Research Consortium (PAN-CRC), College of Human Ecology, Kansas State University, 1105 Sunset Ave, 66502 Manhattan, KS USA

**Keywords:** Postprandial lipemia, Inflammation, Aerobic exercise, High-fat meal, Physical activity

## Abstract

Postprandial lipemia is an independent risk factor for development of cardiovascular disease. Postprandial inflammation following the prolonged elevation of triglycerides occurring subsequent to ingestion of high-fat meals, provides a likely explanation for increased disease risk. Substantial evidence has shown that acute exercise is an effective modality for attenuation of postprandial lipemia following a high-fat meal. However, much of the evidence pertaining to exercise intensity, duration, and overall energy expenditure for reducing postprandial lipemia is inconsistent. The effects of these different exercise variables on postprandial inflammation is largely unknown. Long-term, frequent exercise, however, appears to effectively reduce systemic inflammation, especially in at-risk or diseased individuals. With regard to an acute postprandial response, without a recent bout of exercise, high levels of chronic exercise do not appear to reduce postprandial lipemia. This review summarizes the current literature on postprandial and inflammatory responses to high-fat meals, and the roles that both acute and chronic exercise play. This review may be valuable for health professionals who wish to provide evidence-based, pragmatic advice for reducing postprandial lipemia and cardiovascular disease risk for their patients. A brief review of proposed mechanisms explaining how high-fat meals may result in pro-inflammatory and pro-atherosclerotic environments is also included.

## Background

The typical Western diet is characterized by sizable portions of highly processed foods, large amounts of added sugars, and a high total fat content. The average fat content of a Western meal is between 20 and 40 g, and three to four meals per day are consumed regularly [1]. Therefore, many individuals spend the majority of their day in a postprandial state, characterized by elevated levels of circulating triglycerides (TRG) following a meal. As Western diet patterns have become commonplace in developed countries around the world, atherosclerotic related deaths have also increased [2]. Extensive research over the last several decades has focused on one of the main components of the Western diet, the high-fat content, and its role in the development of atherosclerosis.

Throughout much of the 20^th^ century, atherosclerosis was thought to be merely a disease of excess lipids in the bloodstream. However, recent evidence has shown the progression of the disease to be more related to inflammation within the blood vessel wall [3]. Accordingly, individuals with elevated levels of systemic inflammation have been shown to have an increased risk for sudden cardiac events and mortality [4–6]. Previous research suggests that even a single high-fat meal (HFM) may induce postprandial inflammation [7, 8] and endothelial dysfunction [9], due to the prolonged elevation of TRG in the blood stream known as postprandial lipemia (PPL) [10]. Although there is no widely agreed upon definition of PPL, postprandial lipemia may be defined as the prolonged elevation of TRG and triyglyceride-rich lipoproteins (TRLs) in circulation following the consumption of a meal. A prolonged elevation of TRG allows for high-density lipoproteins (HDL) to be cleared from the blood stream more easily [11], and for small, atherogenic low-density lipoprotein (LDL) particles to form [12].

Evidence suggests that PPL may be attenuated through acute bouts of exercise as well as chronic aerobic training [13, 14]. Furthermore, highly active individuals tend to have lower levels of systemic inflammation as compared to less active individuals [15]. However, the benefits of acute exercise for postprandial lipemia appear to be relatively short lived. Only a few days without exercise may completely negate any attenuation of PPL following the last bout of exercise [16]. Due to the short-term effects of acute exercise on PPL, researchers have examined how exercise timing, type, and intensity, may impact the lipid lowering effects of exercise [17]. The majority of these studies have focused on TRG clearance from circulation; however, how TRG clearance effects the relationship between acute exercise and postprandial inflammation is not well understood. Understanding how exercise effects circulating TRG is important due to the pro-inflammatory and pro-atherosclerotic environment resulting from prolonged elevations of TRG. However, postprandial TRG must be examined along with other physiological processes occurring during exercise that may affect the postprandial inflammatory environment.

In this review we will discuss the postprandial inflammatory response to high-fat, high-calorie, Western style meals, and the roles of both acute and chronic exercise as methods of attenuating PPL and inflammation. A brief review of proposed mechanisms explaining how high-fat meals may result in pro-inflammatory and pro-atherosclerotic environments will also be included.

## Methods

### Data sources

The primary database that was searched was PubMed with minor contributions from studies found via Google Scholar.

### Key words

All of the following key words were used in different combinations in an attempt to cover the most thorough breadth of the research as possible. These terms include “postprandial” or “post-prandial”, “post-meal”, “lipemia” or lipaemia”, “triglycerides”, “exercise”, “aerobic”, “resistance”, “high-fat meal”, “inflammation”, “inflammatory”, “cytokines”, “atherosclerosis”, “heart disease”.

### Selection of studies

For studies that discussed postprandial lipemia, the dependent variable had to be some measure of circulating triglycerides in the time period following ingestion of a meal. Unless otherwise noted, studies in which participants performed exercise were primarily moderate-intensity aerobic forms of exercise. Additionally, studies were selected that focused on healthy, normal lipemic participants except for when a different population is mentioned within the context of a referenced study. We had few parameters for the selection of studies within a given section of the paper. The authors gave the most weight to the studies that had been most commonly cited in the literature and were most up to date. The goal of this review is to provide a narrative examination of studies that follows the majority of the weight of evidence for each section. Studies that provide contrasting evidence are also acknowledged.

## Postprandial hyperlipidemia

The magnitude of the postprandial lipid increase is directly proportional to the fat content of a meal up to approximately 80 g [18], and many metabolic processes determine the overall magnitude and duration of PPL. Recently digested lipids must be absorbed and secreted in the form of chylomicrons from the small intestine, and very low-density lipoproteins (VLDL) are secreted from the liver. Hereafter, we will refer to chylomicrons and VLDLs jointly as TRLs in circulation.

**Fig. 1:**
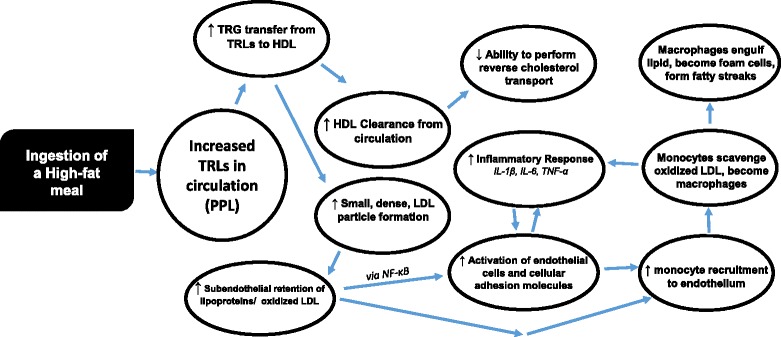
The proposed pro-inflammatory pathway following ingestion of a HFM. Triglycerides (TRG), Triglyceride Rich Lipoproteins (TRL), Postprandial lipemia (PPL), High-density lipoprotein cholesterol (HDL), Low Density Lipoprotein cholesterol (LDL), Nuclear Factor Kappa B (NF-κB), Interleukin 1β (IL-1β), Interleukin-6 (IL-6), Tumor Necrosis Factor-α (TNF-α). Increased TRG in circulation leads to increase HDL clearance from circulation and an increase in small dense LDL particles. This process increases subendothelial retention of lipoproteins, leads to oxidized LDL, and activation of the vascular endothelium. The activated endothelium increases the recruitment of immune cells, mainly monocytes, to the vascular surface to scavenge oxidized LDL molecules. Accumulation of lipid inside monocytes leads to formation of macrophages and secretion of several pro-inflammatory cytokines. Macrophages accumulate enough lipid to form foam cells that result in fatty streaks within the vascular endothelium

Elevated plasma TRLs in the postprandial state are an independent risk factor for coronary heart disease [19]. The mechanisms through which TRLs cause damage to the vascular wall are not fully understood; however, the following proposed mechanisms may help explain the increased cardiovascular disease risk associated with elevated postprandial TRLs (as illustrated in Fig. 1). Increased TRLs in circulation lead to a greater transfer of triglyceride from TRLs to cholesterol-rich lipoproteins (mainly HDL) via cholesterol ester transfer protein [12]. This process both depletes HDL [20] and increases concentrations of small, dense, LDL and chylomicron (CM) remnants [21]. These small, dense lipoprotein remnants appear to have less LDL-receptor binding affinity than large LDL particles [22], allowing them to stay in circulation longer and become atherogenic through their ability to penetrate the vascular endothelium [23].

## Postprandial inflammatory response

Once the small, dense CM and LDL particles have penetrated the vascular endothelium, they are oxidized by reactive oxygen species (ROS) [24]. Monocytes are recruited to the endothelial surface by increased expression of vascular cell adhesion molecule-1 (VCAM-1) [25]. VCAM-1 expression may be increased via oxidized LDL particles that may activate the nuclear factor kappa B (NF-kB) pathway [26]. Monocytes penetrate the endothelial wall and become macrophages as they scavenge oxidized lipoproteins, as these macrophages accumulate lipid they become foam cells [27]. This buildup of foam cells leads to a fatty streak within the blood vessel [28]. This process is the initiation of an atherosclerotic plaque.

A variety of cytokines are secreted from macrophages that contribute to the progression of atherosclerosis. Many of these cytokines, such as interleukin-6 (IL-6) and tumor necrosis factor- alpha (TNF-α), lead to the production of downstream cytokines and various other inflammatory markers that play vital roles in the regulation of atherosclerotic plaques [29]. Although there are dozens of biomarkers that effect the development of atherosclerosis, we have chosen to focus on a few biomarkers have been shown to play prominent roles in the development of atherosclerotic plaques. These markers include IL-6, TNF-α, interleukin 1β (IL-1β), C-reactive protein (CRP), and the cellular adhesion molecules sVCAM-1 and intercellular adhesion molecule-1 (ICAM-1).

A frequently studied biomarker of postprandial inflammation is IL-6. IL-6 is secreted from endothelial cells, T cells and macrophages within the vascular endothelium, as well as from adipose tissue [30]. In the context of the postprandial period, IL-6 is generally considered to be pro-inflammatory [31]. Evidence has shown that increased levels of IL-6 are correlated with greater occurrence of cardiac events [32]. IL-6 also plays an important role in the synthesis of CRP by the liver [33] and regulation of TNF-α [34]. Several studies have shown increases in IL-6 in the postprandial period following high-fat, high-calorie meals [35–37], while others have not [38, 39]. Studies that have shown increased IL-6 in the postprandial period, typically have had participants consume higher total fat and calorie meals than those that have shown no increase in IL-6. More recently, IL-6 has also been recognized as a myokine, because of its secretion from skeletal muscle tissue during exercise [40]. Its role as a myokine is generally considered to be anti-inflammatory, and will be discussed in detail later in this review.

Along with IL-6, TNF-α is another commonly measured pro-inflammatory biomarker. TNF-α is produced by macrophages, endothelial cells and adipose tissue [41, 42]. TNF-α has been shown to induce the expression of leukocyte cellular adhesion molecules such as VCAM-1 and ICAM-1 [43]. Additionally, TNF-α may also enhance the production of other inflammatory cytokines [44]. The direction and magnitude of TNF-α responses in the postprandial period have been mixed. TNF-α may increase postprandially in overweight participants [37], and in participants with metabolic syndrome [45]. However, other studies have reported no change [38, 46] or even a small decrease [35] in TNF-α postprandially. These conflicting results are most likely due to differences in participant characteristics between studies. For example, TNF-α concentrations may be increased in participants with greater abdominal adiposity and/or advanced age [47, 48].

CRP is one of the classic biomarkers used to assess systemic inflammation. Elevated levels of CRP have been shown to be associated with a number of chronic cardiovascular diseases including coronary heart disease, hypertension, and stroke [49]. CRP is of particular interest in the postprandial period because of its downstream response to increases in IL-6 [50], its controversial role in atherosclerotic plaques [51, 52], and specifically in the recruitment of monocytes to the arterial intima [53]. However, the exact role of CRP in the development of atherosclerosis is still unclear. It has been well established that elevated levels of CRP indicate an increased risk for the development of CVD, but the question of whether or not CRP has a causal role in the development of CVD still remains unclear [54]. It is possible that CRP may only be a marker of inflammation in atherosclerotic plaques resulting from acute increases in IL-6 and TNF- α [55]. A review by Herieka and Erridge (2013) indicated only one out of 30 studies measuring CRP in the postprandial period found an increase in the acute phase protein in the immediate hours following a HFM [56]. Any significant increases in circulating CRP following a HFM may be found closer to 24 h after ingestion of the meal after acute increases in IL-6 and TNF- α have already occurred in the inflammatory cascade.

Interleukin-1β (IL-1β) is a member of the IL-1 family that has been implicated in nearly all aspects of the atherosclerotic process. IL-1β may be present in monocytes and macrophages [57], as well as adipose tissue [58]. When secreted, IL-1β may lead to the downstream production of other inflammatory markers such as IL-6 and CRP [59]. IL-1β has been shown to promote a hyper-triglyceridemic and pro-atherogenic lipid profile through the increased production and decreased clearance of VLDLs [60]. Additionally, the pro-inflammatory environment caused by IL-1β may promote structural changes in LDL particles that result in the smaller, denser, LDLs that can more easily penetrate the endothelium and be oxidized [61, 62]. Furthermore, IL-1β may be secreted from macrophages when they engulf oxidized LDLs, which would continue stimulation of the inflammatory/pro-atherogenic environment. IL-1β can directly initiate the development of atherosclerotic plaques through the recruitment of cellular adhesion molecules, facilitating the migration of additional inflammatory cells to the vascular endothelium [63]. IL-1β does not appear to increase after a high-fat meal in healthy subjects [64, 65], but may increase in individuals with metabolic syndrome [66].

A key aspect of the initial stages of atherosclerosis is the adhesion of inflammatory cells, mainly leukocytes, to the vascular endothelium. Two important adhesion molecules shown to have implications in the development of atherosclerosis are VCAM-1 [67] and ICAM-1 [68]. These molecules are measured in the blood after they have been detached from the endothelium and are circulating in their soluble form. VCAM-1 and ICAM-1 are expressed on the endothelial surface of the blood vessel wall [69] and upregulated in response to increased concentrations of several pro-inflammatory cytokines [70]. These adhesion molecules enable the adhesion and penetration of leukocytes across the vascular endothelium [69]. Some evidence suggests that increases in soluble adhesion molecules may only occur in an already pro-atherosclerotic environment [71], such as in diabetic [72] and hypertriglyceridemic [73] individuals. Additionally, both ICAM-1 and VCAM-1 have been reported to predict future cardiovascular events in patients with coronary artery disease [5]. Further, postprandial increases in cellular adhesion molecules in healthy subjects have been shown to be small [8] or nonexistent [74, 75].

## Assessment of PPL and postprandial inflammation

Currently, there is no firmly established protocol for HFM testing to assess PPL. However, most studies that have experimentally assessed PPL have used similar procedures with minor variations. The first consideration when assessing PPL is the controlling of confounding lifestyle variables leading up to the testing session. Exercise is well known to decrease the PPL response; it is usually prohibited for at least 48–60 h prior to the test meal [13]. Caffeine is also typically excluded for ~24 h prior to the HFM test. Diet is obviously a very important factor to control leading up to a PPL session. The extent to which diet is controlled varies across studies, ranging from tightly controlled studies that provide meals for multiple days [76], to studies that only require participants to log food their food intake and/or keep meals consistent between trials [46]. However, even more important than controlling diet is the requirement of ensuring that individuals fast (for at least 8 h, but typically 10–12 h) prior to their test meal. Metabolic effects of a single meal can linger for hours, and an individual’s metabolic state at the onset of a PPL study will affect their postprandial response. Once in the lab, a baseline blood draw is performed. In most studies, a cannula is inserted in a forearm vein to allow for easier repeated samples [76–78], although some studies do utilize repeated venipuncture [8, 79]. Following the baseline/fasting blood draw, the participant will consume the test meal. Test meals vary greatly between studies – one reason that comparisons across studies can be very difficult. Meals typically contain >40% fat and >700 kcal, with some containing more than >60% fat and >1500 kcal [80]. Most study designs normalize the meal to each participant’s body weight, so that the amount of kcals consumed per kilogram of body mass or lean body mass is the same for each participant [80]. After the meal is consumed, blood draw procedures are repeated for a given duration at a set frequency. Most studies assess the PPL response for four [78] to eight hours postprandially [76]. These blood draws are used to assess several metabolic markers, with triglycerides as the primary analyte assessed in most PPL studies. However, there are other metabolic markers that are occasionally assessed that can provide additional information regarding the postprandial metabolic state. Most of these additional markers are apolipoproteins or lipoprotein subfractions, including: total cholesterol, HDL, LDL, apolipoprotein C3 (ApoC3), which is found on several types of lipoproteins of intestinal and hepatic origin; apolipoprotein B100 (ApoB100), which is found on VLDL, IDL, and LDL; and remnant cholesterol.

## Chronic exercise and PPL

It has been well established that regular exercisers have a lower postprandial lipemic response compared to non-exercisers [14, 81]. The triglyceride lowering effects of frequent exercise are often attributed to increased HDL concentrations [20], increased post-exercise lipoprotein lipase (LPL) activity [82], and replenishment of intramuscular triglycerides (IMTG) post exercise [83]. In healthy participants, previous research has indicated that there is an inverse relationship between HDL-C and PPL following a HFM [20]. Additionally, participants who completed a jogging program 3-days/week displayed significantly higher plasma LPL concentrations when compared to their own control condition at baseline [84]. The combination of higher HDL-C and LPL concentrations in regularly exercising individuals may allow for greater replenishment of IMTG and improved clearance of TRG following exercise. Consistent upregulation of triglyceride clearing mechanisms may help lower the mean residence time of LDL in circulation. A shorter residence time allows less time for more of the small, dense, LDL particles to form and pose an increased risk for atherosclerosis [85].

The benefits of exercise training, however; may be short lived even in the most active individuals [86]. For example, one study found that after 60 h without exercise, there was no difference in PPL between trained and untrained subjects. This study compared exercise trained individuals (half endurance trained, half sprint trained), to individuals who participated in no more than two, 30-min exercise sessions per week. Evaluations that occurred at least 60 h following their last bout of exercise indicated that there were no significant differences in postprandial triglycerides between endurance-trained, strength-trained, and untrained participants [86]. Physiological adaptations to detraining, such as reduced aerobic capacity, occur over a much longer time period than 60 h [87]. The effects of exercise on intramuscular triglyceride replenishment and LPL activity appear to be absent at 60 h post exercise. Therefore, mechanisms independent of aerobic capacity are the likely drivers of exercise attenuated PPL.

Kiens and Richter (1998) investigated the effects of exercise training on lipid metabolism in endurance-trained males [83]. Their study focused on IMTG use during post-exercise recovery and the possible role of LPL in this process. The authors found that IMTG stores were diminished post-exercise until 42 h. LPL activity was significantly increased the day following exercise, but had returned to baseline levels at 42 h post-exercise. These data suggest that circulating TRLs could be broken down by increased LPL activity, used to replenish IMTG stores, and provide fuel for post-exercise recovery [83]. When LPL activity is returned to baseline levels and IMTG stores are replenished, the attenuating effect of previous exercise on PPL appears to be absent. In combination, LPL activity and IMTG storage may help explain why the lipid-lowering effects of an exercise training program appear to be so transient.

Despite the apparent brevity of several of the PPL-lowering adaptations from exercise, there does appear to be some benefit of prior training status with regard to PPL. In a study performed by Tsetsonis, Hardman, and Mastana (1997), trained and untrained (VO_2_ max 50.3 ± 5.9, 31.7 ± 3.6 ml/kg/min respectively) middle-aged women completed a bout of exercise of similar duration and intensity (90 min at 60% VO_2_ max), 16 h prior to ingestion of a high-fat meal. There was no significant difference in lipemia between groups after their non-exercise control sessions, but trained women had a 30% reduction in lipemia when compared to untrained women the morning following the exercise session [88]. However, since the relative exercise intensity of each group was the same, but the aerobic capacity of the trained group was higher, the trained group achieved a greater overall energy expenditure. Greater energy expenditures achieved during shorter durations of exercise performed at the same relative exercise intensity for trained subjects as compared to untrained subjects, provide a potential explanation for the lower PPL responses in trained subjects. The attenuated PPL response in trained participants may also be due to upregulated triglyceride clearing mechanisms and enhanced ability to use fat for fuel during exercise (increased mitochondrial and capillary density perhaps) over untrained subjects.

## Acute exercise, timing and PPL

Exercise performed during the timeframe between 18 h pre-meal, until around 90 min post-meal may be effective in attenuating PPL. Gill et al. (2004) showed that exercise in the 12–18 h range prior to ingestion of a high-fat meal attenuated the lipemic response to a high-fat meal [89]. Additionally, Zhang et al. (2004) showed an attenuation of PPL in participants that exercised 12 h, but not 24 h prior to a high-fat meal [90]. However, Johnson et al. (2015) found that exercise 12 h prior to a HFM was ineffective for attenuating PPL even though Johnson et al. and Zhang et al. both had participants exercise at 60% VO_2_ max for 60 min [46]. Additionally, exercise performed immediately before ingestion of a HFM has also been shown to be effective for attenuating PPL [90, 91]. Collectively, this evidence reinforces the idea that pre-meal exercise benefits for PPL are relatively acute and are highly dependent on energy expenditure and participant characteristics. Pre-meal exercise beyond 18–24 h prior to a HFM, likely has little effect on PPL. In addition to pre-meal exercise, evidence also supports exercise during the post-meal period as an effective means for attenuation of PPL, the majority of work in this area has suggested that exercise performed as far as 90 min into the postprandial period is effective for attenuating PPL [92, 93], however this evidence is conflicting [77, 78].

The mechanisms for exercise-induced attenuation of PPL may differ slightly depending on the timing of the relationship between the bout of exercise and the ingested meal. Exercise performed well in advance of the meal (12-18 h) may attenuate PPL via increased LPL expression on the surface of the vascular endothelium [94]. LPL expression in response to exercise appears to increase between 4–8 h post-exercise [95], returning to baseline levels around 20 h post-exercise [96], which may explain the lack of PPL attenuation when exercise is performed 24 h or more before the meal. Exercise performed immediately before the meal may attenuate PPL through a decreased hepatic secretion of VLDLs [97]. Prolonged exercise is thought to reduce fatty acid synthesis, increase fatty acid oxidation and cause accumulation of TRG in the liver in response to prolonged exercise. It has also been shown that circulating VLDL concentrations are decreased at 4.5 h post-exercise under post-absorptive conditions [98]. Bellou et al. 2013 showed that women decreased the TRG content of their VLDL by 30% the morning after an exercise session. These women decreased both their hepatic TRG secretion and increased their TRG clearance; the latter likely due to increased LPL activity, although LPL was not measured [99]. Overall it appears that for exercise performed before a high-fat meal, PPL attenuation is initially due to decreased hepatic VLDL secretion, and as more time passes between the exercise bout and meal ingestion, LPL activity gradually becomes a more prominent factor leading to greater TRG clearance from circulation. However, Sondergaard et al. 2011 did not find an increase in TRG clearance during or following exercise, but rather, a slight nonsignificant decrease in TRG clearance during both time periods [97]. These contrasting results may be partially due to differences in exercise intensity and energy expenditure between studies. Participants in the Bellou et al. 2013 study exercised at 60% VO_2_ max for an average of 123 min, while participants in the Sondergaard et al. 2011 study exercised at 50% VO_2_ max for 90 min. These constrasting findings highlight the difficulty in accurately measuring and interpreting lipid kinetics during exercise, especially when there are differences in study design [97, 99].

**Fig. 2:**
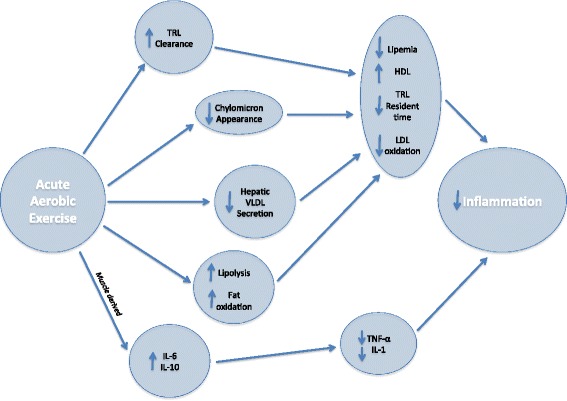
The proposed anti-inflammatory effect of acute aerobic exercise. Triglyceride rich lipoproteins (TRL), High-density lipoprotein cholesterol (HDL), Low-density lipoprotein cholesterol (LDL), Very-low density lipoprotein cholesterol (VLDL), Interleukin-1 (IL-1), Interleukin-6 (IL-6), Interleukin-10 (IL-10), Tumor Necrosis Factor- α (TNF- α). Acute aerobic exercise decreases circulating triglycerides through increased TRL clearance from circulation, decreased TRL appearance from the small intestine, decreased VLDL secretion from the liver, and increased lipolysis and fat oxidation. Each of these processes contributes to lower PPL, increased HDL, decreased TRL resident time in circulation, and decreased LDL oxidation. These processes combine to create a less inflammatory environment within the vasculature. Muscle contractions during aerobic exercise release the anti-inflammatory cytokines IL-6 and IL-10. These cytokines inhibit production of pro-inflammatory cytokines TNF- α and IL-1 leading to lower inflammation within the vasculature

PPL lowering mechanisms for post-meal exercise appear to be similar to that of pre-meal exercise. A small but significant amount of circulating TRG may contribute to total energy expenditure during exercise; however, the absolute rate of VLDL oxidation during exercise is not different compared to resting conditions [97]. Additionally, greater blood flow through the vasculature during exercise leads to increased contact time between TRG and LPL and more opportunity for TRG to be hydrolyzed. Lastly, in a similar manner to pre-meal exercise, post-meal exercise may decrease hepatic fatty acid synthesis and reduce VLDL accumulation in the bloodstream [97]. (The mechanisms for exercise-induced attenuation of PPL are included in Fig. 2).

## Exercise energy expenditure, intensity and energy balance and PPL

### Energy expenditure

Exercise frequency, intensity, duration, and energy expenditure are all important considerations for a complete exercise program. When specifically targeting attenuation of PPL, overall energy expenditure may be the most influential factor to consider. In a previous review by Petitt and Cureton (2003), the authors concluded that the magnitude of prior exercise induced energy expenditure appears to directly determine the magnitude of PPL attenuation [100].

Previous research has attempted to determine a threshold for energy expenditure needed to attenuate PPL. Researchers have shown energy expenditures in excess of 1000 kcal can effectively attenuate PPL [13, 101]. However, studies using such large energy expenditures are not representative of an energy expenditure that is realistic for most individuals to achieve during regular bouts of exercise. Energy expenditures of this magnitude are most likely only achieved by highly trained individuals with aerobic capacities exceeding that of the general population. However, some evidence suggests that obese individuals and those with lower aerobic capacities may require less exercise energy expenditure to attenuate PPL. In less aerobically fit men, relatively low energy expenditures both prior to, and post-exercise have been shown to attenuate PPL. The pre-meal exercise bout used in one study resulted in an energy expenditure of less than 500kcals [102]. A separate study found a postprandial walk of only 30 min was also effective for attenuating PPL [103]. Other studies that have tested recreational exercisers have shown that these individuals may require energy expenditures in the 600-900 kcal range in order to attenuate PPL [104, 105]. Therefore, it appears that individuals who exercise less frequently may need to expend less energy during exercise to attenuate PPL. However, individuals who exercise more frequently and have higher aerobic capacities, may require a higher energy expenditure to attenuate PPL.

### Intensity

Exercise intensity is highly related to exercise energy expenditure, and previous research has explored the question of what exercise intensity is required for a reduction in PPL. Low intensity exercise performed the evening before (15 h prior) a high-fat meal has been shown to be effective for attenuating PPL [106]. However, to achieve an adequate energy expenditure to attenuate PPL at a low-intensity, this bout of exercise took 120 min to complete, which is a duration that may be unattainable for most individuals on a daily basis. Further, when low-intensity exercise was performed for only 90 min, no attenuation of PPL was shown [101]. Moderate-intensity exercise, however, has been shown to attenuate PPL with a duration more representative of typical exercise habits. Zhang et al. (1998) showed that 60 min of moderate-intensity exercise (60% VO_2_ max) was effective for attenuating PPL when performed one hour or 12 h prior to a HFM [91]. To further support the benefits of moderate-intensity exercise, two previous studies have indicated that as little as 30 min of brisk walking at a moderate-intensity can be effective for attenuating PPL [102, 107]. Both of these studies compared continuous exercise to shorter, intermittent bouts and found both types of exercise to be equally effective. Furthermore, prior high-intensity exercise has also been found to be an effective modality for reducing PPL following a HFM [17]. This effect has been show through both cycling and treadmill running [108, 109]. Additionally, the benefits of high-intensity exercise have also been shown in youth populations, where higher exercise intensities are more likely to be achieved than in adult populations [110, 111]. A recent quantitative review by Freese et al. 2014, compared high-intensity exercise to low-to-moderate intensity aerobic exercise. One of the meta-analyses from that review revealed that high-intensity exercise induced a greater attenuation of PPL (*d* = 1.49) as compared to aerobic exercise at lower intensities (*d* = 0.58) [17]. Collectively, it appears that high-intensity exercise prior to a HFM may be advantageous as compared to lower-intensity exercise in regard to time and PPL attenuation per amount of energy expended. However, due to the strenuous nature of higher-intensity exercise, it should be recommended that individuals exercise at an intensity level that is safe for them individually, especially in high-risk populations.

Although post-meal exercise has been less frequently studied compared with pre-meal exercise, similar exercise intensities appear to be beneficial for both timeframes. Moderate-intensity exercise in the postprandial period has been shown to be effective for attenuating PPL [93]. However, low-intensity exercise with comparable timing has produced mixed results; some researchers have shown lower-intensity exercise to be effective for attenuating PPL [92], others have not [112]. However, similar to pre-meal exercise, low-intensity exercise was only effective for attenuation of PPL after a long 90 min bout of exercise [93]. At the present time, little evidence regarding high-intensity exercise in the postprandial period is available, possibly due to gastrointestinal distress that may occur if high-intensity exercise is performed immediately following a HFM. Overall, it appears that moderate-to-high intensity aerobic exercise, may be the most effective for attenuating PPL. However, considerations must be made for each individual’s health, exercise capabilities, and available time before engaging in strenuous exercise, particularly in the immediate timeframe around a HFM.

### Energy balance

Prior exercise has been consistently shown to be an effective way to attenuate PPL [81, 113], however, the relationship between overall energy balance, exercise, and PPL is still not fully understood. In one study, when participants replaced 110% of the calories expended during exercise with a meal replacement drink, the triglyceride lowering effects of a bout of exercise were no longer present [114]. In a different study where participants performed long, exhaustive bouts of exercise. Participants either remained in negative energy balance, or replaced oxidized carbohydrates with a high-glycemic-load drink. Participants who consumed the carbohydrate replacement beverage following exercise did not have an attenuated PPL response [115]. However, Chiu et al. (2014) found reduced TRG concentrations the morning following moderate-intensity exercise even after post-exercise glucose replacement [116]. Contrasting results between studies may be due to different exercise intensities effecting the energy substrate used during exercise. Also, different participant characteristics (mainly age and obesity status) may affect participants’ ability to metabolize energy replacement carbohydrates.

The comparative roles of diet-induced versus exercise-induced energy deficit has also been investigated. Using a novel study design, Maraki et al. (2010), examined energy deficit through dietary restriction alone, exercise induced, or a combination of both. Results indicated that all three groups had an attenuated PPL response, with the exercise induced energy deficit group achieving the greatest attenuation [117]. These findings are in agreement with a study by Gill and Hardman (2000) that found a non-significant reduction in postprandial triglycerides by energy restriction alone, but an exercise induced energy deficit to be three times as effective as energy restriction alone [118]. Maraki and Sidossis (2010) reviewed the effect of negative energy balance on PPL and concluded that for aerobic exercise to be effective for reducing PPL, an energy deficit of around 7 kcal/kgbw is required [119]; this equates to about 60 min of moderate-intensity exercise or 120 min of low-intensity exercise. Collectively, it appears an energy restrictive diet and an energy deficit created through exercise may both contribute to reductions in PPL, however exercise induced energy deficits may result in a greater magnitude of reduction.

## Resistance exercise and PPL

The majority of previous research on exercise and PPL has employed low-to-moderate intensity aerobic exercise, but there is also supporting evidence indicating that prior acute resistance exercise may also attenuate PPL [120, 121], however this evidence is not conclusive [122]. A direct comparison of the PPL lowering effects of resistance exercise to aerobic exercise showed that acute resistance exercise was as effective at reducing PPL in women [123]. In this study, women completed separate trials of 60 min of moderate aerobic exercise and 60 min of resistance training with similar resultant attenuation of PPL even though the resistance exercise bout likely provided a lower total energy expenditure than the aerobic exercise [123]. In regard to exercise intensity, it does not appear that the PPL attenuation is any different following moderate vs. high-intensity resistance exercise when total exercise volume remains constant [121]. Additionally, increased exercise volume and total energy expenditure during an acute bout of resistance exercise does not result in greater attenuation of PPL the morning after exercise, when total energy balance remains constant [124]. These findings reinforce the major role that total energy balance plays in the relationship between all forms of exercise (aerobic and resistance) and PPL.

## Exercise and PPL in youth populations

Risk factors for the disease process of atherosclerosis have been shown to begin early in life [125], therefore it is important to examine the potential PPL reducing effects of exercise in youth populations. Tolfrey et al. recently reviewed this topic and found the effects of exercise on PPL to be similar in adult and youth populations [126]. For example, moderate-intensity exercise appears to both lower PPL [127] and improve endothelial function [128] the morning after exercise. Notably, the positive effects of intermittent exercise and high-intensity interval training, may be especially advantageous in youth, due to the often spontaneous nature of youth exercise and an increased ability to handle high-intensity exercise bouts as compared to adult populations [110, 129, 130]. Youth populations should be encouraged to meet physical activity guidelines and engage in high-intensity exercise when possible. Meeting such guidelines will likely reduce the likelihood of developing atherosclerotic lesions that may be present in some youth populations [131].

## The anti-inflammatory effect of acute exercise

Inflammatory cytokines during and immediately after exercise can be difficult to measure and achieve consistent results. However, previous literature supports an anti-inflammatory effect for exercise, even when studied acutely. As an example, IL-6 has been extensively studied as both a pro- and anti-inflammatory cytokine. IL-6 has be shown to increase nearly exponentially (up to 100 fold) when released from contracting skeletal muscle during exercise [132]. IL-6 has also been shown to increase in response to greater exercise intensity and duration. When released from skeletal muscle, IL-6 appears to be anti-inflammatory and has been shown to increase lipolysis and fat oxidation without an increase in circulating TRG [133]. Increased IL-6 production during exercise has an inhibitory effect on both TNF-α and IL-1 [134] and increases production of the anti-inflammatory cytokine IL-10 [134]. Subsequently, IL-10 has been shown to inhibit the production of many pro-inflammatory cytokines including: IL-1β, TNF-α, and [135]. Together these mechanisms could explain the anti-inflammatory effects of IL-6 secreted by skeletal muscle during exercise. (The anti-inflammatory effect of acute aerobic exercise is included in Fig. 2).

## Chronic exercise and systemic inflammation

As more research has established atherosclerosis as an inflammatory disease, many studies have examined the relationships between long-term exercise habits, aerobic fitness, and systemic inflammation. Highly active individuals typically display lower levels of circulating systemic inflammation, compared to low-active individuals [136, 137]. In a study by Fischer et al. (2007), eighty-four healthy adults were divided into four groups based on presence or absence of obesity and their typical physical activity level [138]. The authors found elevated levels of IL-6 and CRP in inactive individuals to be independent risk factors for systemic inflammation even after controlling for obesity, age, gender, and smoking status. In agreement with these findings, epidemiological evidence suggests an inverse relationship between physical activity and several pro-inflammatory biomarkers, including IL-6 and TNF-α [139]. However, exercise intervention trials have been less conclusive with regard to the beneficial effects of frequent exercise on inflammation markers. Results from one meta-analysis revealed a non-significant three percent reduction of CRP among exercise intervention trials lasting a minimum of four weeks [140]. Beavers et al. (2010), reviewed the effects of exercise interventions on systematic inflammation and found 6 out of 12 studies reduced inflammation, 5 of which included CRP. Exercise intervention studies that include participants with high levels of systematic inflammation due to obesity or a chronic disease, are more likely to show reductions in systemic inflammation during the course of an exercise intervention than studies with healthy participants who already have low levels of inflammation [141]. Thus, it appears that the short-term effectiveness of exercise interventions for reducing inflammation may largely depend on participant characteristics at baseline, but it has been consistently shown that patterns of frequent exercise in the long-term appear to result in lower levels of systemic inflammation [15, 140]. Dixon et al. (2009) performed a study to examine whether or not higher levels of long-term physical activity resulted in a lower postprandial inflammatory response. Participants were split into two groups: active participants were those who participated in greater than 90 min of vigorous activity per week and 30 min of moderate activity 5 days/week; inactive subjects engaged in no vigorous activity and 30 min of moderate activity less than 5 days/week. Results indicated that active subjects had lower postprandial glucose, insulin, and TRG concentrations, but there were no differences in postprandial inflammatory markers between groups [136]. Based on these data, it is evident that frequent exercise is moderately effective for reducing long-term systemic inflammation, but this relationship does not appear to effect the acute inflammatory response following a HFM. It is possible that the role of exercise as an anti-inflammatory agent may be more related to long-term adaptations to frequent bouts of exercise that result in physiological changes in the vasculature and skeletal muscle at the cellular level. Increased capacity to metabolize lipids for fuel and an increased anti-oxidant capacity in more aerobically fit individuals may result in an improved ability to handle large metabolic loads and maintain lower levels of chronic, systematic inflammation when compared to less frequent exercisers wither lower levels of aerobic fitness.

## Resistance exercise and inflammation

The majority of studies examining exercise and the inflammatory response have focused on endurance aerobic exercise, but there is evidence to suggest an inflammatory effect from acute resistance exercise as well. Similarly to endurance exercise, IL-6 appears to increase between 3 and 6 h after a resistance exercise session, albeit to a lesser magnitude than after endurance exercise [142]. Additionally, TNF- α does not appear to increase following acute resistance training [143] and may even decrease [144]. Evidence of long-term effects of resistance exercise on resting inflammatory markers appears to be weaker than evidence for acute effects. For example, in one study that included a 12-week resistance training program, resting levels of inflammatory cytokines were not lowered, with the exception of CRP [145]. However, it does appear that resistance trained individuals have upregulated mRNA expression of anti-inflammatory cytokines such as IL-6 and IL-10 as compared to non-resistance trained individuals [146].

## Acute exercise and postprandial inflammation

The effects of an acute bout of exercise on postprandial inflammation are relatively unknown. Experimental studies aimed at reducing postprandial inflammation through exercise have focused primarily on moderate-to-high intensity pre-meal exercise [104, 105]. While these studies have found prior exercise to be effective for attenuating PPL, they have not found acute exercise to be effective for decreasing postprandial inflammation. Other mechanisms, in addition to PPL may be responsible for the pro-inflammatory environment seen following a HFM. Experimental studies have examined the relationship between prior exercise and postprandial markers of oxidative stress and endothelial function [109, 147]. These studies have shown that bouts of moderate and high-intensity exercise may be able to diminish or prevent increases in oxidative stress and endothelial dysfunction caused by a HFM. This may be due to an increased anti-oxidant capacity during exercise. Increased ROS during exercise may signal to the muscle to increase antioxidant capacity. Muscle cell adaptations to reduce ROS during exercise may also result in an overall protection against ROS at rest [148]. Overall, it appears that moderate to high-intensity aerobic exercise prior to a HFM is effective for attenuating PPL, oxidative stress, and endothelial function. However, studies that have used acute bouts of exercise prior to a HFM have not provided any evidence to indicate beneficial effects on postprandial pro-inflammatory cytokine concentrations.

## Future directions

As opposed to focusing exclusively on exercise prior to a HFM, future studies should also directly examine postprandial exercise as a potential modality to reduce both postprandial inflammation and lipemia. Additional focus should be placed on more true-to-life investigations into the interaction between diet and exercise as they related to postprandial lipemia and inflammation. Meal sizes proportional to the body size of participants; exercise of realistic duration, intensity, and total volume; and meals and exercise occurring in a time window when individuals typically eat before or after exercise should be investigated further. Evidence from such investigations could provide more pragmatic information for health professionals to provide to clients who are trying to improve their blood lipid profile or reduce overall CVD risk.

## Conclusion

Acute exercise from 18 h prior to a HFM until 90 min after a HFM has been shown to be effective in attenuating PPL. Exercise at a moderate-intensity for a 60 min duration has been shown in several different studies to attenuate PPL, this is a recommendation that may be achievable as compared to bouts of low-intensity exercise of 120 min duration. Although 60 min of moderate exercise has been shown to be effective for reducing PPL, studies thus far have not demonstrated that an acute bout of exercise is effective for attenuating postprandial inflammation. Many studies, however, have indicated that long-term frequent exercisers have lower basal systemic inflammation as compared to less frequent exercisers. Yet, without the presence of a recent bout of exercise, high levels of chronic exercise do not appear to reduce PPL. It may be practical for health practitioners to prescribe frequent exercise at a moderate-intensity to consistently attenuate PPL and lower systemic inflammation over time. Combined with more moderate sized meal portions, this recommendation could protect individuals from metabolic stress and may result in lower PPL and postprandial inflammation long term.

## Abbreviations

Apo B100: Apolipoprotein B100
ApoC3: Apolipoprotein C3
CM: Chylomicron
CRP: C-Reactive Protein
HDL: High-density lipoprotein
HFM: High-fat meal
ICAM-1: Intercellular adhesion molecule-1
IL-1β: Interleukin 1β
IL-6: Interleukin-6
IMTG: Intramuscular triglycerides
LDL: Low-density lipoprotein
LPL: Lipoprotein lipase
NF-kB: Nuclear factor kappa B
PPL: Postprandial lipemia
ROS: Reactive oxygen species
TNF- α: Tumor necrosis factor-alpha
TRG: Triglycerides
TRL: Triglyceride-rich lipoproteins
VCAM-1: Vascular cell adhesion molecule-1
VLDL: Very low-density lipoprotein
